# Synthesis and Optimization of Next-Generation Low-Molecular-Weight Pentablock Copolymer Nanoadjuvants

**DOI:** 10.3390/vaccines11101572

**Published:** 2023-10-09

**Authors:** Alaric C. Siddoway, Brianna M. White, Balaji Narasimhan, Surya K. Mallapragada

**Affiliations:** 1 Department of Chemical & Biological Engineering, Iowa State University, Ames, IA 50011, USA; asiddo@iastate.edu (A.C.S.); bmwhite@iastate.edu (B.M.W.); nbalaji@iastate.edu (B.N.); 2 Nanovaccine Institute, Ames, IA 50011, USA

**Keywords:** polymer, adjuvant, nanomaterial, block copolymer, vaccine, antigen delivery

## Abstract

Polymeric nanomaterials such as Pluronic^®^-based pentablock copolymers offer important advantages over traditional vaccine adjuvants and have been increasingly investigated in an effort to develop more efficacious vaccines. Previous work with Pluronic^®^ F127-based pentablock copolymers, functionalized with poly(diethyl aminoethyl methacrylate) (PDEAEM) blocks, demonstrated adjuvant capabilities through the antigen presentation and crosslinking of B cell receptors. In this work, we describe the synthesis and optimization of a new family of low-molecular-weight Pluronic^®^-based pentablock copolymer nanoadjuvants with high biocompatibility and improved adjuvanticity at low doses. We synthesized low-molecular-weight Pluronic^®^ P123-based pentablock copolymers with PDEAEM blocks and investigated the relationship between polymer concentration, micellar size, and zeta potential, and measured the release kinetics of a model antigen, ovalbumin, from these nanomaterials. The Pluronic^®^ P123-based pentablock copolymer nanoadjuvants showed higher biocompatibility than the first-generation Pluronic^®^ F127-based pentablock copolymer nanoadjuvants. We assessed the adjuvant capabilities of the ovalbumin-containing Pluronic^®^ P123-based pentablock copolymer-based nanovaccines in mice, and showed that animals immunized with these nanovaccines elicited high antibody titers, even when used at significantly reduced doses compared to Pluronic^®^ F127-based pentablock copolymers. Collectively, these studies demonstrate the synthesis, self-assembly, biocompatibility, and adjuvant properties of a new family of low-molecular-weight Pluronic^®^ P123-based pentablock copolymer nanomaterials, with the added benefits of more efficient renal clearance, high biocompatibility, and enhanced adjuvanticity at low polymer concentrations.

## 1. Introduction

Multiple efforts have been dedicated to the development of novel vaccine adjuvants and delivery systems, which have the potential to significantly improve the performance of current vaccine technologies [1,2,3,4,5]. Biocompatible polymeric nanomaterial systems represent a particularly promising route to pursue [6,7,8,9]. In particular, self-assembling micellar systems have an ideal combination of small size (10–100 nm) and multiple functional groups, allowing for interactions with both antigens and immune cells to elicit robust immune responses [10,11,12,13,14].

Of these micellar systems, pentablock copolymers synthesized in our laboratory, based on Pluronic^®^ F127 with poly(diethyl aminoethyl methacrylate) (PDEAEM) blocks at the ends, show high biocompatibility and exhibit excellent immune-enhancing characteristics by maintaining antigenicity, eliciting cytokine production, and inducing robust B cell responses and enhanced antibody titers via crosslinking B cell receptors [15,16,17,18,19]. These polymers have been shown to serve as effective nanoadjuvants and carriers for influenza subunit nanovaccines [20] without inducing the production of reactive oxygen species, nitric oxide, or pro-inflammatory cytokines, as is commonly observed with traditional adjuvants [15,18,21,22]. However, these polymers have relatively high molecular weights (14.5 kDa) compared to traditional adjuvants (e.g., toll-like receptor agonists), which may present long-term concerns for renal clearance [23,24,25]. Furthermore, relatively large doses of polymeric nanomaterials (i.e., 12.5 mg/dose) are needed to adjuvant the protein antigens in vaccine formulations, representing a ca. 250-fold mass ratio of polymer to antigen [26]. The potential shortcomings related to polymer dose and molecular weight could be exacerbated from a regulatory perspective, as vaccines based on these materials are evaluated in large animals and eventually in humans.

This work aims to address the above shortcomings of these first-generation pentablock copolymer adjuvants using rational design, via tailoring the synthesis of a new family of biocompatible pentablock copolymers with shorter Pluronic^®^ P123 instead of Pluronic^®^ F127 to constitute the middle blocks of the pentablock copolymer nanomaterials. Pluronic^®^ polymers are triblock copolymers of poly(ethylene oxide)-b-poly(propylene oxide)-b-poly(ethylene oxide) (PEO-b-PPO-b-PEO). Similar to Pluronic^®^ F127 (MW of 12,600 Da, PEO:PPO:PEO 100:65:100), Pluronic^®^ P123 (MW of 5800 Da, PEO:PPO:PEO 20:69:20) is also used as a surfactant in cosmetics, and has been explored as a drug carrier for use in cancer treatment [16,27,28,29,30]. Desolvation of the large hydrophobic core in Pluronic^®^ P123 acts to endothermically drive self-assembly into nanoscale micellar structures at approximately the same concentration as Pluronic^®^ F127 [31,32,33,34,35,36]. Reducing polymer molecular weight is advantageous because Pluronic^®^ based pentablocks are biocompatible, but not biodegradable, and must be renally excreted [24,37]. In this study, we systematically developed a new generation of tailored and optimized biocompatible pentablock copolymer adjuvants by reducing the polymer molecular weight, and determined the composition and amount of pentablock copolymer required to effectively adjuvant protein antigen(s). We also evaluated the biocompatibility and adjuvanticity of these low-molecular-weight polymeric nanomaterials.

## 2. Materials and Methods

### 2.1. Materials

For polymer synthesis, N,N-(diethylamino)ethyl methacrylate (DEAEM), polyvinylpyrrolidone (MW = 40,000 Da), and pyridine-2-carboxaldehyde were purchased from Sigma Aldrich (St. Louis, MO, USA). Alpha-bromoisobutyryl bromide, basic alumina powder, copper acetate monohydrate, diethyl ether, magnesium sulfate, methylene chloride, n-propylamine, pentane, Pluronic^®^ F127, Pluronic^®^ P123, sea sand, sodium borohydride, triethylamine, and toluene were purchased from Fisher Scientific (Waltham, MA, USA).

### 2.2. Pentablock Copolymer Synthesis

#### 2.2.1. Preparation of Copper (i) Oxide Nanoparticles

The pentablock copolymers were synthesized via atom transfer radical polymerization (ATRP) using modified Pluronic^®^ triblocks as macroinitiators. ATRP uses copper catalysts, and preparation of copper-free pentablock copolymers is dependent on the substitution of ionic copper with ionic copper nanoparticles, which can be more easily removed via filtration or chromatography [15,38]. Copper (i) oxide nanoparticle synthesis began with the preparation of a 20 mMol solution of sodium borohydride in nanopure water. 0.8 wt% of polyvinylpyrrolidone (40 kDa) was added; then, a 2.2 mMol solution of cupric (ii) acetate was prepared and added directly to the sodium borohydride and polyvinylpyrrolidone solution, and an immediate change in color was observed. The reaction was allowed to proceed for six hours before centrifugation at 15,000 rcf. The resulting particle pellet was washed with water, resuspended, and recentrifuged three times. The pellet was collected and dried overnight in a vacuum chamber. The resulting pellet was massed and stored over desiccant under a dried nitrogen atmosphere until use. The final yield was highly dependent on the storage conditions and purity of the sodium borohydride precursor. The reaction was observed to scale linearly, with the only notable safety consideration being the production of hydrogen gas when the two solutions were mixed.

#### 2.2.2. Preparation of NPPM Ligand

Preparation of N-propylpyridynyl methanimine (NPPM) ligand for the ATRP reaction began with the addition of 60 mL of diethyl ether, 60 mL of 2-pyridine carboxaldehyde and 25 g of anhydrous magnesium sulfate to a 250 mL round-bottomed flask. The mixture was chilled on an ice bath while mixing. The flask was sealed with a rubber septum and subjected to three cycles of degassing for 5 min under vacuum, alternating with flushing by dry nitrogen for 5 min. Then, 87 mL of n-propylamine was added, the ice bath was removed, and the mixture allowed to warm to room temperature, where the reaction then proceeded for two hours.

The reaction mixture was filtered to remove the insoluble magnesium sulfate. Diethyl ether was then removed under rotary evaporation. The solution was distilled twice under vacuum, collecting only the solution transferred between 90 °C and 110 °C. The resulting solution was a light blue oil, with a yellow-gold color developing on exposure to oxygen. The resulting NPPM was stored under dry nitrogen until use. Its effectiveness as a ligand was observed to be dependent on storage conditions, with the yellow-gold to orange-colored NPPM indicating unspecified degradation.

#### 2.2.3. Synthesis of Macroinitiators and the ATRP Reaction

**Scheme 1:**
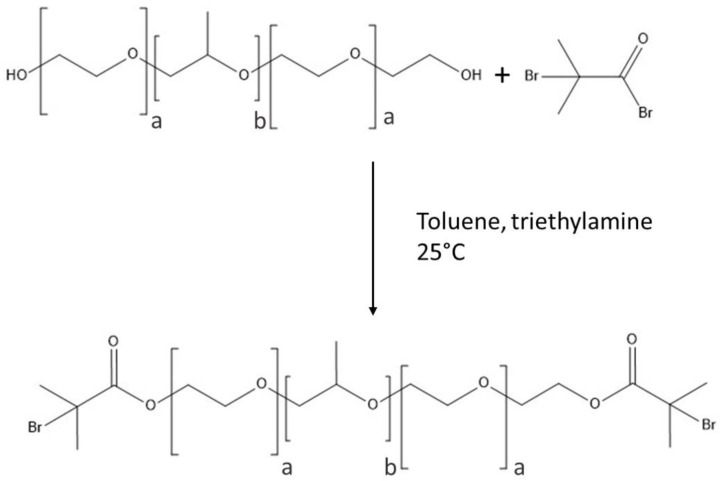
Synthesis of a brominated Pluronic^®^ macroinitiator from a Pluronic^®^ block copolymer for ATRP synthesis of PDEAEM-P123-PDEAEM or PDEAEM-F127-PDEAEM. For Pluronic^®^ F127, a = 100, b = 65. For Pluronic^®^ P123, a = 39, b = 69.

Brominated Pluronic^®^ P123 or Pluronic^®^ F127 was used as macroinitiators for the ATRP reaction. Difunctional 2-bromo-propionate Pluronic^®^ P123 or Pluronic^®^ F127 was prepared as shown in Scheme 1 by adding 20 mMol of Pluronic^®^ P123 or Pluronic^®^ F127 to 100 mL of toluene in a Schlenk flask. Some 10 mL of trimethylamine and 2 mL of α-bromoisobutyryl were added, and the reaction was left overnight at room temperature.

**Scheme 2:**
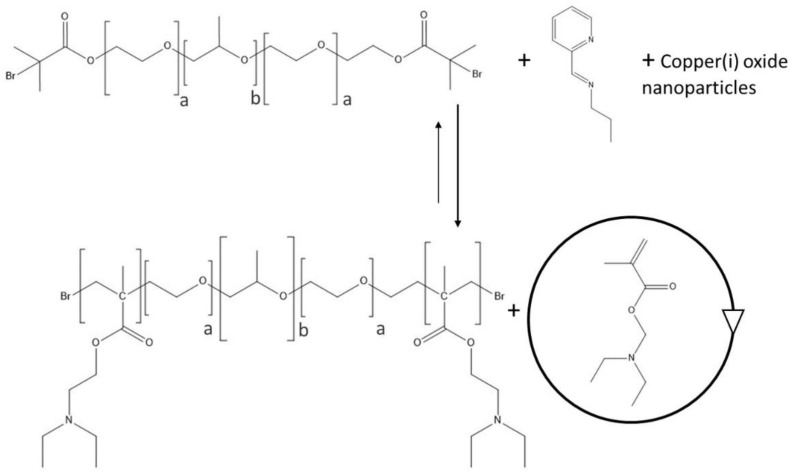
Synthesis of PDEAEM-P123-PDEAEM by ATRP.

PDEAEM-P123-PDEAEM copolymer was prepared directly using the macroinitiation reaction mixture, as shown in reaction Scheme 2. The reaction had developed a dark amber color when 0.25 g of copper(i) oxide nanoparticles and 4 mL of DEAEM were added, and the flask was then sealed with a rubber stopper. Using a Schlenk line, the flask was degassed under a vacuum and flushed with dry nitrogen three times, for five minutes at each step. The mixture was further degassed via three freeze-pump-thaw cycles. Finally, 0.5 mL of NPPM was added to the mixture via syringe and needle, and the ATRP reaction was carried out under agitation at 70 °C. The tailored synthesis of polymers differing by one DEAEM unit per tail was achieved by controlling the reaction time and allowing the reaction to proceed for longer periods of time to increase PDEAEM lengths, as measured by NMR, and is summarized in Table 1. Reactions were halted by removing the rubber stopper, exposing the rection to oxygen, and adding 10 mL of isopropyl alcohol.

To purify the polymer product, the reaction mixture was diluted with an equal volume of methylene chloride and passed over a basic alumina column. The product was collected as an amber solution from the column and concentrated via rotary evaporation and dried under vacuum overnight. The final product was a viscous oily liquid, and was stored over desiccant until use.

### 2.3. NMR and GPC Characterization

All ^1^H NMR spectra were collected using a Varian VXR400 (400 MHz) spectrometer, and chemical shifts are presented as ppm. Chloroform-d was used as a solvent for all ^1^H NMR measurements.

Gel permeation chromatography (GPC) measurements utilized a Waters 2690 separation module with a Waters 2414 refractive index detector and a Waters 2996 photodiode array detector. Molecular weights and polydispersity were calculated relative to polystyrene standards.

### 2.4. Micelle Characterization via DLS and Zeta Potential

Dynamic light scattering (DLS) and the zeta potential of the nanoscale micelles were measured using a ZetaSizer Nano (Malvern Analytical, Malvern, UK). A range of aqueous polymer solutions at a neutral pH were prepared, from 10,000 μg/mL to 10 μg/mL. This concentration range included the critical micellar concentrations for both Pluronic^®^ F127 (35 μg/mL) and Pluronic^®^ P123 (25 μg/mL), as published in the literature [15,36].

### 2.5. Antigen Release Kinetics

Endotoxin-free ovalbumin (OVA) was purchased from InVivogen (San Diego, CA, USA). The release kinetics of OVA from the pentablock copolymer micelles was investigated by preparing three replicate 1 mL polymer solutions containing 100 mg/mL of polymer with 250 μg/mL of OVA in sterile PBS (pH 7.2). Samples were incubated at 37 °C, and protein release was initiated with the addition of 500 μL of PBS (pH 7.2). Periodic samples were taken by removing the supernatant layer above the polymer-gel layer and replacing it with fresh PBS. Protein content was determined using a micro-bicinchoninic acid (BCA) assay (Pierce, Rockford, IL, USA), as described previously [26]. Sampling continued until no further protein was released.

### 2.6. Biocompatibility Studies

ASPC-1 cells (ATCC, Manassas, VA, USA), which are human-derived pancreatic cancer cells, were cultured in Roswell Park Memorial Institute (RPMI) media supplemented with 10% fetal bovine serum and 1% penicillin/streptomycin. Cultures were incubated at 37 °C and 5% CO_2_. Cell culture materials were purchased from Fisher Scientific, and the MTT dye was purchased from Invitrogen. Cell viability was assessed using an MTT assay. ASPC-1 cells were seeded in 96-well plates at a density of 8000 cells/well and incubated overnight. After 24 h, cells were treated with pentablock copolymers dissolved in PBS at various concentrations. The cells were incubated with polymer solutions for 48 h. After incubation, 3-(4,5-dimethylthiazol-2-yl)-2,5-diphenyltetrazolium bromide (MTT) dye was added to the media at a final concentration of 0.5 mg/mL and then incubated for 3 h. Media containing the dye was removed and stop solution (1:1 DMSO to methanol) was added to dissolve the formazan crystals. After the crystals were fully dissolved, the absorbance of the solution was measured at 570 nm using a SpectraMax M3 plate reader (Molecular Devices, San Jose, CA, USA).

### 2.7. In Vivo Studies

All animal procedures were performed in accordance with the Guidelines for Care and Use of Laboratory Animals of Iowa State University, and approved by the Iowa State University Institutional Animal Care and Use Committee.

Five to seven-week-old female C57BL/6 mice were purchased from Jackson Laboratories (Bar Harbor, ME) and used for investigating the adjuvant properties of PDEAEM_5_-F127-PDEAEM_5_, DEAEM_1_-P123-DEAEM_1_, and PDEAEM_2_-P123-PDEAEM_2_. The subscripts indicate the number of DEAEM units attached to the Pluronic^®^ backbone. Similar to previous work with PDEAEM-F127-PDEAEM with OVA [26], groups of mice were immunized (n = 4 per group) with a 100 μL subcutaneous injection consisting of either an aqueous polymer solution (concentration ranging from 50,000 μg/mL to 0.5 μg/mL) with 25 μg OVA, 25 μg OVA alone, or saline, using a prime-boost regimen. Vaccine formulations are summarized in Table 2. Immunizations occurred on days 0 and 28, and 50 μL of blood was collected on days 21 and 42 to determine antibody responses.

### 2.8. Antibody and Avidity Responses

Antibody titers were determined via an enzyme-linked immunosorbent assay (ELISA) with horseradish peroxidase-linked secondary antibodies. Blood drawn from mice was centrifuged at 5000 rcf for 15 min. Sera were separated and stored at 4 °C until use, and at −80 °C afterwards. High-binding, flat-bottom 96-well plates were coated with 100 μL 0.5 μg mL^−1^ OVA in pH 9.6 carbonate/bicarbonate buffer at 4 °C overnight. Wells were blocked with 2% (*w*/*v*) gelatin in PBS with 0.05% Tween-20 (PBS-T) for two hours at room temperature. Plates were washed three times in PBS-T before adding 100 μL of PBS-T to each well. Sera samples were added as the primary antibody in a 1:201 ratio, with 1:2 serial dilutions across the plate. The primary antibody was incubated at 4 °C overnight. Plates were again washed three times in PBS-T before adding 100 μL of PBS-T with secondary horseradish peroxidase-linked secondary antibody at a 1:20,000 ratio. Plates were incubated for two hours at room temperature. Plates were again washed three times in PBS-T followed by the addition of 75 μL ultra-TMB-ELISA substrate solution (Thermo Fisher, Waltham, MA). The reaction was allowed to proceed for 20 min and halted by the addition of 75 μL of 2M sulfuric acid, whereupon the optical density (450 nm) was recorded. For analysis, the background was defined as the average optical density of the saline administered mice. Titer was defined as the reciprocal of the last dilution with an optical density greater than two times the background.

Antibody avidity was determined via ELISA, as described by Sato et al. [39]., with sera diluted 1:201, without serial dilutions. Following overnight primary antibody incubation, an additional incubation with 6M urea was performed for 5 min prior to secondary antibody addition. The avidity index was defined as the ratio between the absorbance of the urea-treated samples and the absorbance of the untreated samples.

### 2.9. Statistical Analyses

Data are presented and compared as a median and standard deviation of three replicates, unless otherwise noted. All analyses were performed using GraphPad Prism 9. (GraphPad Software, Inc., San Diego, CA, USA). For the reaction kinetics analysis, a simple linear regression was performed. Statistical differences in ELISA data were calculated using nonparametric Kruskal–Wallis and Mann–Whitney tests, and p-values less than 0.05 were considered significant.

## 3. Results

### 3.1. Synthesis and Characterization of Pentablock Copolymers

This work aimed to develop and synthesize the next generation of a family of Pluronic^®^-based pH and temperature-responsive self-assembling pentablock copolymer nanoadjuvants. Previous work has demonstrated the versatility of pentablock systems to simultaneously deliver and adjuvant protein payloads to elicit B cell responses [15,18,26].

**Figure 1:**
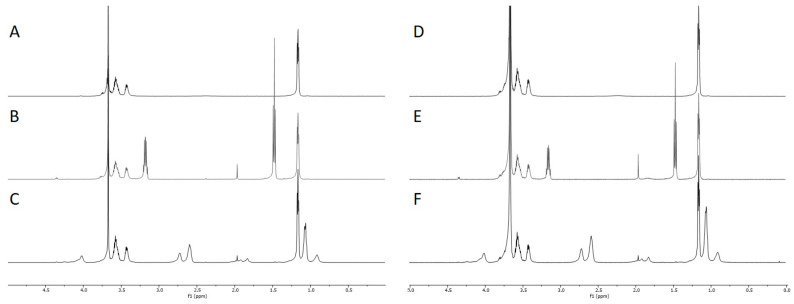
^1^H NMR spectra for Pluronic^®^ P123 (**A**), Pluronic^®^ P123 macroinitiator (**B**), PDEAEM_5_-P123-PDEAEM_5_ (**C**), Pluronic^®^ F127 (**D**), Pluronic^®^ F127 macroinitiator (**E**), and PDEAEM_5_-F127-PDEAEM_5_ (**F**) in deuterated chloroform.

Previous synthesis of the Pluronic^®^ F127-based pentablock copolymers involved a separate preparation of difunctionalized brominated macroinitiatior followed by the ATRP reaction [15]. In this work, a new one-pot, two-step reaction was developed, with samples taken from macroinitiation reactions to confirm the conversion of hydroxyl end groups to bromide end groups (using ^1^H NMR), as shown in Scheme 1. Notably, the ^1^H NMR spectra of the reaction mixture showed significant signals from triethylammonium bromide, indicative of macroinitiation on the Pluronic^®^ backbone (Figure 1). ^1^H NMR spectra of both Pluronic^®^ F127 and Pluronic^®^ P123 macroinitiators were comparable, with additional triethylammonium bromide peaks present at δ 3.12 and δ 1.92 in both spectra. ^1^H NMR F127 macroinitiator (CDCl_3_) δ 3.63 (s, 800H), δ 3.52 (m, 130H), δ 3.37 (m, 65H), δ 1.43 (s, 12H), δ 1.12 (m, 168H). ^1^H NMR P123 macroinitiator (CDCl_3_) δ 3.61 (s, 312), δ 3.51 (m, 138H), δ 3.37 (m, 69H), δ 1.90 (s, 12H), δ 1.10 (m, 207H).

**Figure 2:**
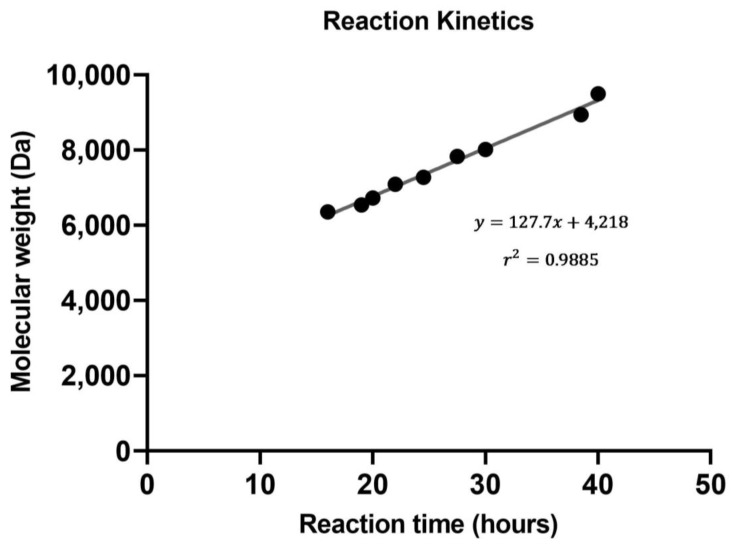
Reaction kinetics of PDEAEM-P123- PDEAEM ATRP. Linear regression found the reaction to be zero order.

ATRP was then carried out, as shown in Scheme 2, with the additional reactants in the macroinitiation reaction mixture. To determine the ATRP reaction kinetics, synthesis under a wide range of reaction durations was performed, with the molecular weights of the resulting polymers summarized in Table 1. Plotting reaction duration against resultant polymer weight clearly shows zero-order reaction kinetics (Figure 2) for this controlled radical polymerization reaction. ^1^H NMR (CDCl_3_) for PDEAEM_5_-F127-PDEAEM_5_ copolymer (Figure 1). δ 3.97 (s, 20H), δ 3.62 (s, 800H), δ 3.52 (m, 130H), δ 3.38 (m, 65H), δ 2.67 (m, 20H), δ 2.55 (m 40H), δ 1.77 (m, 20H), δ 1.11 (m, 195H), δ 1.01 (m, 60H), δ 0.87 (s, 30H). ^1^H NMR (CDCl_3_) for PDEAEM_5_-P123- PDEAEM_5_ copolymer δ 3.97 (s, 20H), δ 3.62 (s, 312H), δ 3.52 (m, 138H), δ 3.37 (m, 69H), δ 2.67 (m, 20H), δ 2.54 (m 40H), δ 1.78 (m, 20H), δ 1.11 (m, 207H), δ 1.01 (m, 60H), δ 0.87 (s, 30H).

Analysis via GPC was limited by the solubility of the polymers in chloroform. Pluronic^®^ P123 pentablocks with more than two DEAEM subunits per tail displayed significantly reduced solubility in chloroform and were excluded from further testing. The polydispersity of DEAEM_1_-P123-DEAEM_1_ and PDEAEM_2_-P123-PDEAEM_2_ pentablock copolymers was determined via GPC and found to be 1.0237 and 1.0235, respectively, with molecular weights corresponding to the ^1^H NMR analysis.

### 3.2. Micelle Characteristics

**Figure 3:**
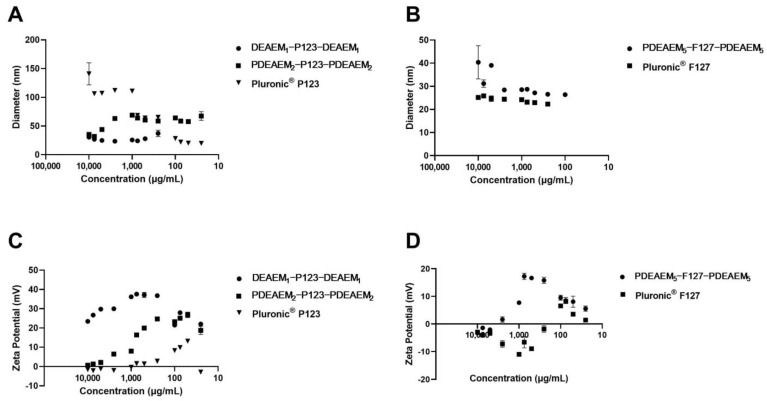
Micelle size measured by DLS and zeta potential. (**A**) Size of DEAEM_1_-P123-DEAEM_1_, PDEAEM_2_-P123- PDEAEM_2_, and Pluronic^®^ P123 micelles as a function of polymer concentration in water. DEAEM_1_-P123-DEAEM_1_ samples below 250 μg/mL were unable to be resolved. n = 3 for each sample, error bars represent standard deviation. (**B**) Size of PDEAEM_5_-F127- PDEAEM_5_ and F127 micelles as a function of polymer concentration in water. F127 samples below 250 μg/mL were unable to be resolved, PDEAEM_5_-F127- PDEAEM_5_ samples below 100 μg/mL were unable to be resolved. n = 3 for each sample, error bars represent standard deviation. (**C**) Zeta potential of DEAEM_1_-P123-DEAEM_1_, PDEAEM_2_-P123-PDEAEM_2_, and Pluronic^®^ P123 micelles as a function of polymer concentration. n = 3 for each sample, error bars represent standard deviation. (**D**) Zeta potential of PDEAEM_5_-F127-PDEAEM_5_ and Pluronic^®^ F127 micelles as a function of polymer concentration. n = 3 for each sample; error bars represent standard deviation. Error bars may be obscured by symbol size.

To determine the micellization characteristics of the newly synthesized polymers in water, a range of aqueous polymer solutions from 10,000 μg/mL to 10 μg/mL were prepared with the previously characterized PDEAEM_5_-F127-PDEAEM_5_ pentablocks and the new DEAEM_1_-P123-DEAEM_1_ and PDEAEM_2_-P123-PDEAEM_2_ pentablock copolymers. Pluronic^®^ P123 pentablocks with more than two DEAEM subunits per tail displayed significantly decreased solubility in water and were excluded from further testing. The solutions were then characterized by DLS, followed by zeta potential measurements (Figure 3). Micellar structures were unable to be detected at concentrations below 250 μg/mL in Pluronic^®^ F127 and DEAEM_1_-P123-DEAEM_1_ solutions, below 100 μg/mL in PDEAEM_5_-F127-PDEAEM_5_ solutions, and below 25 μg/mL in Pluronic^®^ P123 and PDEAEM_2_-P123-PDEAEM_2_ solutions.

**Figure 4:**
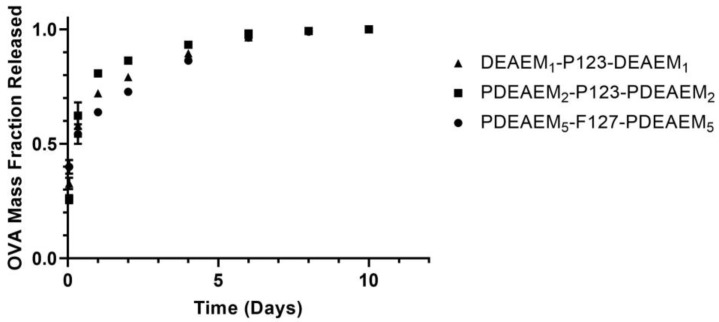
Release kinetics of OVA from DEAEM_1_-P123-DEAEM_1_, PDEAEM_2_-P123-PDEAEM_2_ and PDEAEM_5_-F127-PDEAEM_5_ copolymers. n = 3 for each sample; error bars represent standard deviation. Error bars are present for each time point, but may be smaller than the symbol.

Having characterized the self-assembly of the PDEAEM-P123-PDEAEM pentablocks into micelles, the next step was to measure protein release from the micelles. To this end, DEAEM_1_-P123-DEAEM_1_ and PDEAEM_2_-P123-PDEAEM_2_ polymer solutions with OVA were prepared and incubated at 37 °C. Periodic samples were taken by removing the supernatant from the submerged polymer layer, and protein content was determined via BCA, as shown in Figure 4. Release kinetics were observed to be approximately first-order, occurring over ten days, and were comparable to previous work, with protein release from DEAEM_1_-P123-DEAEM_1_ and PDEAEM_2_-P123-PDEAEM_2_ occurring slightly faster than from PDEAEM_5_-F127-PDEAEM_5_ [26].

### 3.3. Biocompatibility

**Figure 5:**
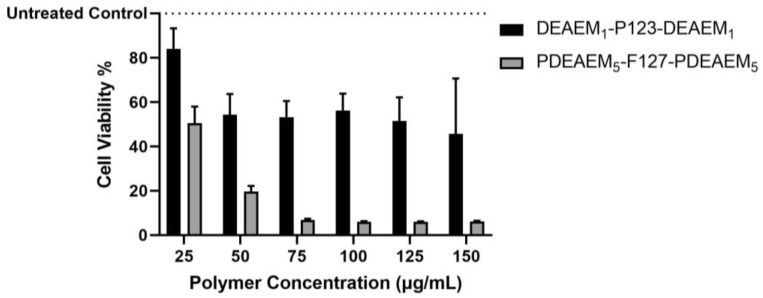
MTT cytotoxicity assay with ASPC-1 cells incubated for 48 h with either PDEAEM_5_-F127-PDEAEM_5_ or DEAEM_1_-P123-DEAEM_1_ groups at various concentrations. Error bars represent the standard deviation, n = 8. The dotted line represents the untreated control wells used to determine percent cell viability.

To determine polymer biocompatibility, ASPC-1 cells were incubated for 48 h with serial dilutions of DEAEM_1_-P123-DEAEM_1_ and PDEAEM_5_-F127-PDEAEM_5._ The cells showed significantly reduced toxicity upon DEAEM_1_-P123-DEAEM_1_ treatment compared to treatments based on the previously synthesized PDEAEM_5_-F127-PDEAEM_5_ (Figure 5). Notably, cell viability in the presence of the new DEAEM_1_-P123-DEAEM_1_ copolymers was above 80% at low polymer concentrations, and approximately 50% at high concentrations, generally outperforming first-generation PDEAEM_5_-F127-PDEAEM_5_ in biocompatibility by a factor of 6 at the higher polymer concentrations.

### 3.4. Antibody Responses

**Figure 6:**
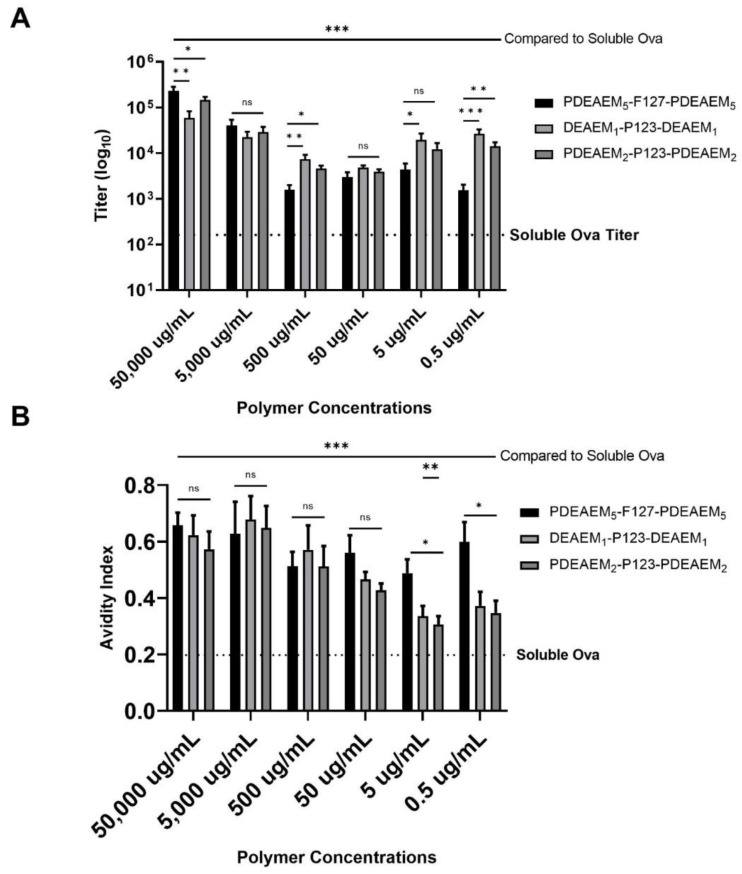
(**A**) Day 42 anti-OVA IgG titers in the sera of vaccinated animals following prime-boost immunization. Titers in all groups were significantly elevated over the titers induced by soluble OVA, as shown by the dashed line. PDEAEM_5_-F127-PDEAEM_5_ groups were compared with DEAEM_1_-P123-DEAEM_1_ and PDEAEM_2_-P123- PDEAEM_2_ groups at each concentration; statistical differences are denoted above each concentration grouping. Statistical differences were determined using Kruskal–Wallis and Mann–Whitney tests. Data are depicted as the mean, with error bars representing the standard error of the mean. 0.0001 < *** < 0.001, 0.001< ** < 0.01, * < 0.05. n = 4 (**B**) Day 42 anti-OVA antibody avidity indexes, representing the absorbance of urea-treated wells over the absorbance of untreated wells. Antibody avidity in the sera of all micelle-adjuvanted groups was significantly elevated over the antibody avidity elicited by soluble OVA, shown by the dashed line. PDEAEM_5_-F127-PDEAEM_5_ treatment groups were compared with DEAEM_1_-P123-DEAEM_1_ and PDEAEM_2_-P123- PDEAEM_2_ at each concentration; statistical differences are denoted above each concentration grouping. Data are depicted as the mean, with error bars representing the standard error of the mean. 0.0001 < *** < 0.001, 0.001< ** < 0.01, * < 0.05. n = 4 with technical replicates, except for PDEAEM_5_-F127-PDEAEM_5_, which had an n = 4 without technical replicates due to the limited sample.

Previous work with Pluronic^®^ F127-based pentablock copolymer micelles has demonstrated adjuvant capabilities through antigen presentation and B cell receptor crosslinking [15,26]. To compare the adjuvant capabilities of the new DEAEM_1_-P123-DEAEM_1_ and PDEAEM_2_-P123-PDEAEM_2_ with the first-generation PDEAEM_5_-F127-PDEAEM_5_ copolymers, groups of mice were immunized with a 100 μL subcutaneous injection consisting of a polymer solution with 25 μg of OVA, or 25 μg of OVA alone, or saline. The treatment groups are summarized in Table 2. Immunizations occurred on day 0 and day 28. Sera were collected at days 21 and 42. No significant anti-OVA antibody titers were detected in sera from any group at day 21, with all samples falling below the limit of detection. By day 42, antibody titers were measurable in all groups with OVA (Figure 6A). Sera from animals immunized with the micelle-adjuvanted OVA produced significant anti-OVA antibody titers at all examined concentrations, compared to the titers in sera from animals immunized with soluble OVA. Antibody avidity was also determined (Figure 6B), and the data indicated that regardless of polymer type or polymer concentration, the avidity of the antibody in the sera of polymer-treated animals was significantly higher than that of the antibody in the sera of animals that received soluble OVA alone.

Notably, sera from animals immunized with the next-generation DEAEM_1_-P123-DEAEM_1_ and PDEAEM_2_-P123-PDEAEM_2_ formulations showed significantly elevated antibody titers even at low concentrations (5 μg/mL and 0.5 μg/mL) compared to both soluble OVA as well as first-generation PDEAEM_5_-F127-PDEAEM_5_ copolymers. Interestingly, while PDEAEM_5_-F127-PDEAEM_5_-based vaccine formulations consistently induced highly avid antibodies, PDEAEM-P123-PDEAEM-based vaccine formulations induced higher antibody titers, with both polymer types showing significant enhancements compared to soluble OVA.

## 4. Discussion

This work aimed to develop a new family of biomaterials based on Pluronic^®^ P123-based pentablock copolymers by replacing the Pluronic^®^ F127 triblock core with a smaller alternate, to expand the range of next-generation pentablock copolymeric adjuvants. In particular, previous work used PDEAEM_5_-F127-PDEAEM_5_ [26] nanoadjuvants, which were ca. 14,500 Da in molecular weight. In addition, large doses of this copolymer (i.e., 12.5 mg/dose) were needed to induce efficacious antibody responses [26]. The significance of the current study is the development of an improved pentablock copolymer adjuvant with a lower molecular weight for effective renal clearance that also requires a lower polymer dose to induce effective antibody responses.

Initial synthesis attempted to produce PDEAEM-P123-PDEAEM with intermediate purification of macroinitiated Pluronic^®^ P123. The reaction was carried out with a direct molar substation of Pluronic^®^ P123 for Pluronic^®^ F127, according to reaction Scheme 1. This produced the desired macroinitiator, but purification proved to be difficult, and was plagued with low yields. In stark contrast to previous work with the Pluronic^®^ F127 macroinitiator, the Pluronic^®^ P123 macroinitiator cannot be precipitated into a crystalline powder. This is not unexpected, because Pluronic^®^ P123 has a significantly lower molecular weight, resulting in a paste “P” morphology at room temperature as compared to F127’s flake “F” morphology. This necessitated the development of a facile one-pot approach for synthesis, with macroinitiation directly leading into ATRP.

Having prepared a macroinitiation reaction mixture with Pluronic^®^ P123 macroinitiator, PDEAEM-P123-PDEAEM copolymers were synthesized, as shown in reaction Scheme 2, under various reaction times to produce copolymers over a range of polymer weights, as shown in Table 1. The reaction times were plotted against the resulting molecular weight and analyzed via simple linear regression (Figure 1). Consistent with previous work, the controlled radical ATRP reaction was observed to follow zero-order kinetics, following a lag activation period [15]. The reaction was notably slower than that observed in previous work with F127-based copolymers, yielding only 2.5 DEAEM units per tail after 20 h of reaction time, whereas the F127 ATRP yielded 5 DEAEM per tail in a similar time frame [15]. This may be due to the increased viscosity observed in Pluronic^®^ P123 ATRP reaction mixtures compared to F127 ATRP reaction mixtures, due to the inclusion of macroinitiation reactants and side products.

Following purification, the resulting product was a viscous liquid. We hypothesize that this is due to the introduction of relatively bulky DEAEM groups, which reduce the ability of individual polymer molecules to pack closely. Molecular weight and purity were confirmed with NMR following macroinitiation and purification (Figure 1). The polydispersity of the polymer product was determined via GPC, with DEAEM_1_-P123-DEAEM_1_ and PDEAEM_2_-P123-PDEAEM_2_ copolymers being essentially monodisperse.

Self-assembly into nanoscale micellar structures plays a major role in the adjuvant properties of these pentablock copolymers [15,26]. Cationic micelles are hypothesized to act as a scaffold, enabling antigen presentation while simultaneously interacting with and crosslinking B cell receptors, thus inducing antibody production [15]. Previous work with PDEAEM-F127-PDEAEM pentablocks established the temperature and pH sensitivity of self-assembly, but did not explore the dependency of geometry and surface charge on concentration [15]. To this end, solutions of Pluronic^®^ P123 and Pluronic^®^ F127 were compared to solutions of PDEAEM-P123-PDEAEM and PDEAEM_5_-F127-PDEAEM_5_. Dynamic light scattering data indicated that P123 micelle size is positively correlated with concentration, with smaller micelles present at lower polymer concentrations (Figure 3). The opposite trend was observed in DEAEM_1_-P123-DEAEM_1_ and PDEAEM_2_-P123-PDEAEM_2_ copolymers, with larger micelles, on average, at lower polymer concentrations. Pluronic^®^ F127 and PDEAEM_5_-F127-PDEAEM_5_ polymer solutions displayed a small decrease in size with decreasing concentration. Micelle size differences at various polymer concentrations were determined to be significantly different via comparison with a *t*-test.

The differences in trends observed in the Pluronic^®^ P123-based pentablock micelles, which were not observed with the F127-based pentablock micelles, may be due to the differences in hydrophobic chain length. Pluronic^®^ P123 and Pluronic^®^ F127 have approximately the same number of core hydrophobic polypropylene oxide subunits; however, Pluronic^®^ P123 only has 78 hydrophilic PEO subunits in comparison to 200 PEO subunits in Pluronic^®^ F127 [16,27]. Therefore, the addition of DEAEM subunits to Pluronic^®^ F127 results in a negligible increase in hydrophilicity, with little disruption of the resultant self-assembly. In contrast, the addition of DEAEM subunits to the much more hydrophobic Pluronic^®^ P123 represents a significant increase in hydrophilicity, and consequently, the self-assembly characteristics, with respect to micelle size and concentration, change accordingly.

Zeta potential was also measured for each polymer sample, and confirmed previous work showing that the functionalization of Pluronic^®^ F127 with DEAEM generated a cationic polymer due to the presence of the DEAEM groups [15,19]. This trend was observed with the new Pluronic^®^ P123-based pentablock copolymers as well (Figure 3). Furthermore, DEAEM_1_-P123-DEAEM_1_, PDEAEM_2_-P123-PDEAEM_2_ copolymers are at least as cationic as PDEAEM_5_-F127-PDEAEM_5_ copolymers, suggesting that DEAEM_1_-P123-DEAEM_1_ and PDEAEM_2_-P123-PDEAEM_2_ may behave similarly in the presence of antigenic protein.

PDEAEM_5_-F127-PDEAEM_5_ copolymers released OVA over a period of one week, which is comparable to previous work (Figure 4) [19]. In contrast, formulations based on DEAEM_1_-P123-DEAEM_1_ and PDEAEM_2_-P123-PDEAEM_2_ exhibited slightly faster antigen release kinetics but were nonetheless comparable to PDEAEM_5_-F127-PDEAEM_5_. This indicates that DEAEM_1_-P123-DEAEM_1_ and PDEAEM_2_-P123-PDEAEM_2_ could be used to deliver proteins on similar timescales as traditional F127-based pentablock copolymers.

Cationic polymers exhibit cytotoxicity resulting from cationic residues interacting with cellular membranes [40]. The first-generation pentablock copolymer, PDEAEM-F127-PDEAEM, was found to be generally biocompatible in both in vitro and in vivo studies, being well tolerated and producing few side effects [17]. Second-generation DEAEM_1_-P123-DEAEM_1_ has been observed to be significantly less cytotoxic (Figure 5), suggesting that DEAEM_1_-P123-DEAEM_1_ would be better tolerated that its predecessor. The reduced cytotoxicity may be as a result of the decreased number of DEAEM subunits present on PDEAEM_2_-P123-PDEAEM_2_, as well as the decreased length of the Pluronic^®^ P123 backbone [40].

Having observed changes in micellar size and surface charge relative to bulk concentration, studies were designed to probe the effect of polymer concentration, as summarized in Table 2. At day 21, no antibody titer to OVA was detected in any group. By day 42, significant anti-OVA antibody titers, with significantly higher avidity as compared to the soluble OVA group, were observed in the sera of vaccinated animals (Figure 6). Mice immunized with all the polymer groups showed significant anti-OVA antibody titers at high concentrations, as expected, consistent with previous work with PDEAEM-F127-PDEAEM pentablocks [26]. Unsurprisingly, decreasing the polymer concentration decreased the resulting antibody titer, though this effect was more pronounced in the PDEAEM_5_-F127-PDEAEM_5_ copolymer groups than in the DEAEM_1_-P123-DEAEM_1_ and PDEAEM_2_-P123-PDEAEM_2_ groups (Figure 6A). Significantly higher titers (19,500 at 5 µg/mL and 26,400 at 0.5 µg/mL for DEAEM_1_-P123-DEAEM_1_; 15,800 at 5 µg/mL and 18,600 at 0.5 µg/mL for PDEAEM_2_-P123-PDEAEM_2_) were elicited by DEAEM_1_-P123-DEAEM_1_ and PDEAEM_2_-P123-PDEAEM_2_ at low concentrations, as compared to those induced by soluble OVA and PDEAEM_5_-F127-PDEAEM_5_. These data indicate that both that DEAEM_1_-P123-DEAEM_1_ and PDEAEM_2_-P123-PDEAEM_2_ could be used at much lower doses. However, both DEAEM_1_-P123-DEAEM_1_ and PDEAEM_2_-P123-PDEAEM_2_ at 5 μg/mL and 0.5 μg/mL significantly underperformed PDEAEM_5_-F127-PDEAEM_5_ at the same concentrations with respect to antibody avidity (Figure 6B). In spite of this, the observed avidity was still significantly higher than that of soluble OVA (0.0001 < *p* < 0.001).

In this work, a 0.05 µg/mL copolymer dose was used to adjuvant 25 µg OVA, representing a 25,000-fold dose reduction compared to previous work with first-generation pentablock copolymers [26]. This low polymer dose could potentially lower vaccine cost, while presenting a relatively palatable option for regulatory agencies. Furthermore, these next-generation Pluronic^®^ P123-based pentablock copolymers have significantly lower molecular weights (DEAEM_1_-P123-DEAEM_1_ MW = 6170 Da and PDEAEM_2_-P123-PDEAEM_2_ MW = 6540 Da) compared to the much larger Pluronic^®^ F127-based pentablock copolymer (PDEAEM_5_-F127-PDEAEM_5_ MW = 14,500 Da). This low molecular weight confers advantages when considering renal clearance [23,37].

It is notable that the very low polymer concentrations used in this work (i.e., 5 µg/mL and 0.5 µg/mL) are below the critical micellize concentration (cmc) of their respective Pluronic^®^ backbones [36] reported in the literature, and below the concentration at which micelles could be detected via DLS (Figure 3). We hypothesize that the shorter DEAEM_1_-P123-DEAEM_1_ and PDEAEM_2_-P123-PDEAEM_2_ copolymers interact with the surface of OVA proteins. Similar to micellization in Pluronic^®^ PEO-PPO-PEO triblocks [31], this process may also be driven by endothermic dehydration of PPO blocks via association with OVA. The cationic tails of DEAEM_1_-P123-DEAEM_1_ and PDEAEM_2_-P123-PDEAEM_2_ copolymers may then reach out from the surface to interact with B cell receptors. Furthermore, while it possible that this process may also occur with PDEAEM_5_-F127-PDEAEM_5_ copolymers, the increased hydrophobicity in DEAEM_1_-P123-DEAEM_1_ and PDEAEM_2_-P123-PDEAEM_2_ copolymer solutions may more effectively facilitate this process. In contrast, the PDEAEM_5_-F127-PDEAEM_5_ copolymer solutions contain longer hydrophilic tails that may wrap around OVA proteins, shielding OVA from interaction with immune cells, thereby reducing the ability of the copolymer micelles to crosslink B cell receptors. This hypothesis may explain the lower anti-OVA titers induced by PDEAEM_5_-F127-PDEAEM_5_ at the lower 5 μg/mL and 0.5 μg/mL concentrations compared to the titers induced by DEAEM_1_-P123-DEAEM_1_ and PDEAEM_2_-P123-PDEAEM_2_.

## 5. Conclusions

In this study, we synthesized and investigated a new family of Pluronic^®^-based pentablock copolymer nanoadjuvants using Pluronic^®^ P123 as the middle block. We report the synthesis and purification of such polymers, along with characterization of micellar size and zeta potential and the dependency of both on polymer concentration. The Pluronic^®^ P123-based pentablock copolymers have higher biocompatibility and much lower molecular weights than the Pluronic^®^ F127-based pentablock copolymers and provide sustained release kinetics of antigens over similar timescales. Finally, we report that when animals are immunized even with very low concentrations of these novel Pluronic^®^ P123 based pentablock copolymers, their sera contain similar enhancement of anti-OVA antibody titers, with similar avidity indexes, compared to those induced by the previously reported Pluronic^®^ F127-based pentablock copolymers at much higher concentrations. In aggregates, these results indicate that both Pluronic^®^ F127-based and Pluronic^®^ P123-based pentablock copolymer adjuvants are able to undergo conformational changes to adjuvant protein antigens at both high and low concentrations. The newly synthesized Pluronic^®^ P123-based pentablock copolymers have the added benefit of significantly reduced polymer molecular weight and polymer dose requirements, potentially facilitating easier renal clearance and lower costs. These characteristics make these novel nanomaterials promising adjuvants in the development of future vaccines.

## Figures and Tables

**Table 1:** PDEAEM-P123-PDEAEM reaction times and molecular weights.

Hours Reacted	Approx. DEAEM Units Per Tail	Molecular Weight (Da)
16.0	1	6170
19.0	2	6540
20.0	2.5	6725
22.0	3.5	7095
24.5	4	7280
27.5	5.5	7835
30.0	6	8020
38.5	8.5	8945
40.0	10	9500

**Table 2:** Copolymers used for immunization of animals with OVA-containing nanovaccines.

Polymer	Polymer Concentrations (μg/mL)	Ovalbumin (μg)
PDEAEM_5_-F127-PDEAEM_5_	50,000	25
PDEAEM_5_-F127-PDEAEM_5_	5000	25
PDEAEM_5_-F127-PDEAEM_5_	500	25
PDEAEM_5_-F127-PDEAEM_5_	50	25
PDEAEM_5_-F127-PDEAEM_5_	5	25
DEAEM_1_-P123-DEAEM_1_	50,000	25
DEAEM_1_-P123-DEAEM_1_	5000	25
DEAEM_1_-P123-DEAEM_1_	500	25
DEAEM_1_-P123-DEAEM_1_	50	25
DEAEM_1_-P123-DEAEM_1_	5	25
PDEAEM_2_-P123-PDEAEM_2_	50,000	25
PDEAEM_2_-P123-PDEAEM_2_	5000	25
PDEAEM_2_-P123-PDEAEM_2_	500	25
PDEAEM_2_-P123-PDEAEM_2_	50	25
PDEAEM_2_-P123-PDEAEM_2_	5	25
Saline	---	25
Saline	---	---

## Data Availability

The data presented in this study are available on request from the corresponding author.
